# Study on the Repair Technology of Laser Damage-Fused Silica Optics Based on the Neural Network Method

**DOI:** 10.3390/ma15155274

**Published:** 2022-07-30

**Authors:** Bo Wang, Wanli Zhang, Feng Shi, Ci Song, Yaofei Zhang, Guoyan Sun, Shuangpeng Guo

**Affiliations:** Laboratory of Science and Technology on Integrated Logistics Support, College of Intelligence Science and Technology, National University of Defense Technology, 109 Deya Road, Changsha 410073, China; wbnudt@163.com (B.W.); shifeng@nudt.edu.cn (F.S.); songci@nudt.edu.cn (C.S.); zhangyaofei18@nudt.edu.cn (Y.Z.); sunguoyan@opt.ac.cn (G.S.); g18231033287@163.com (S.G.)

**Keywords:** fused silica, laser damage, repair, neural network, magnetorheological polishing

## Abstract

As a key component of a high-power laser device, fused silica optics needs to bear great laser energy, and laser damage is easily generated on the optical surface. In order to improve the service life and availability of optics, it is necessary to repair the damaged optics. In this work, the repair technique of damaged, fused silica optics was studied. The neural network method was mainly used to establish the correlation between the number of small-scale damage points and the repair depth. The prediction accuracy of the model is better than 90%. Based on the neural network model, the removal depth parameters were optimized with the suppression coefficient of the damage points. The processing effect of the optimized parameters was verified by magnetorheological polishing experiments. In this paper, a repair technique based on a neural network was proposed, which avoids the low efficiency caused by processing iterations in the repair process, and can accurately what was expected. The method proposed in this work has an important reference value in the repair process of fused silica optics.

## 1. Introduction

A fused silica element is a kind of optical material with excellent properties, which is widely used in the manufacturing process of diffractive elements applied in various laser systems, focusing lenses, fragment shields and other elements in laser devices such as NIF (National Ignition Facility) in the United States and the Shenguang device in China [1,2,3]. Take NIF as an example. In the focusing system, the number of fused silica optics is more than 3000, accounting for about 40% of all optical optics [4]. With the rapid development of high-power laser devices, the demands for fused silica elements are increasing, which put forward higher requirements for the manufacturing capacity of optics.

Despite the continuous improvement of processing techniques of fused silica optics, laser-induced damage has not been improved. Once the initial laser damage occurs, the mechanical, optical, thermal and other properties of optics are changed. Under the subsequent laser irradiation, the size of the damage point may increase rapidly [5,6]. According to the standard of NIF, when the shading area of the damaged point accounts for more than 3% of the surface, it is considered that the service life of the optics is exhausted and they need to be replaced as soon as possible [7]. At the same time, the existence of laser-induced damage will affect the beam transmission quality, and even cause damage to downstream components.

**Figure 1:**
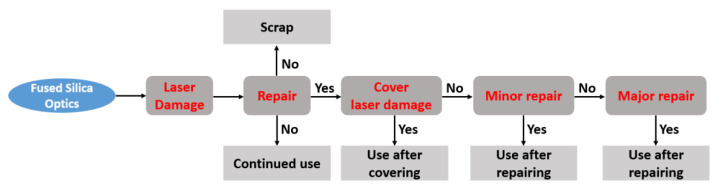
Use flow of fused silica optics.

For high-power laser devices such as NIF, the number of fused silica optics in the system is extremely large, and their processing cycle is long. The method of replacing damaged optics with new ones cannot fully meet the requirements of the device. Therefore, repairing the damaged components is meaningful in maintaining the service performance of high-power laser systems [8,9,10]. Scientists working for the NIF optical system have already carried out the damage repair of optics and put this work in a key position to improve the system performance as shown in Figure 1.

The damage repair methods of fused silica optics mainly include plasma etching, HF (hydrofluoric acid) chemical etching, CO_2_ laser repair, etc. Among them, the CO_2_ laser repair method is used as the main method because of its strong stability [11]. Although the method was proven to inhibit the growth of laser damage, it still has some weaknesses: 1. It can only be applied for damage points in small quantities and large sizes. 2. Low repair efficiency. 3. The optical performance of the repair area becomes invalid after repair, and the repair position cannot continue to guide the beam transmission. For small-scale laser damage, this method is inappropriate. Studies have shown that the probability of damage growth of small-scale laser damage (diameter: <50 μm) is still high under subsequent laser irradiation. When small-scale damage points are repaired, the service life of optics is increased by about 40% [12,13]. It can be seen that small-scale laser damage seriously limits the service life of fused silica optics. Considering the weaknesses of the CO_2_ laser repair method, it is urgent to look for other methods to repair small-scale lasers on the surface of components, so as to improve the service performance of optics and ensure the stable operation of high-power laser devices.

The MRF (Magnetorheological Finishing) technique was developed by Kordonski in cooperation with the center for optics manufacturing (COM) of the University of Rochester in the United States [14]. This technology achieves a material’s removal through high-speed scratch between a flexible polishing ribbon and the surface. The application of a flexible polishing ribbon can avoid introducing new surface damage while removing surface materials [15]. Luo Bin of the GDUT (Guangdong University of Technology) processed sapphire optics using the MRF technique to achieve efficient material removal and avoided introducing new defects [16]. Mahender Kumar Gupta polished fused silica material using the MRF technique, and the optical surface roughness RMS reduced from 0.776 nm to 0.257 nm, which proved that the MRF technique can significantly improve the surface quality of optics [17]. Wenwen Liu passivated the micron defects on the surface of fused silica optics using MRF and chemical etching techniques, and the laser damage threshold of the optics could reach 14 J/cm^2^ [18]. Xiaoyuan Li established the correlation between the stress and surface roughness during the MRF polishing process of fused silica optics and verified that the MRF technique can significantly reduce the surface roughness of components [19]. As an efficient machining method, the MRF technique was used to remove the surface defects and improve the surface quality of fused silica optics. Combined with previous studies, the MRF technique was used to repair small-scale laser damage on the surface of damaged fused silica optics. This repair method can effectively reduce the invalid area of optics without destroying the optical properties at the laser damage area.

In this work, to reduce the repair time and improve the repair efficiency, a neural network method is proposed to predict the repair depth in the MRF process, so as to improve the repair effect of fused silica optics. This work is divided into the following five parts. Besides the Introduction in the first part, the second section is the Method and sample preparation. The third section is the Experimental Results. The fourth section is the Discussion and the fifth part summarizes the research.

## 2. Materials and Methods

### 2.1. Sample Preparation

Several fused silica optics (Supplier: Likabao Co., Ltd., Chengdu, China, material: Heraeus 312, size: φ 50 mm × 10 mm, polishing by conventional polishing technique) were prepared for the experiment. The surface roughness RMS of the optics was better than 1 nm, and the surface profile accuracy PV was better than 1/4 wavelength, which was consistent with the optics used in the Shenguang device.

**Figure 2:**
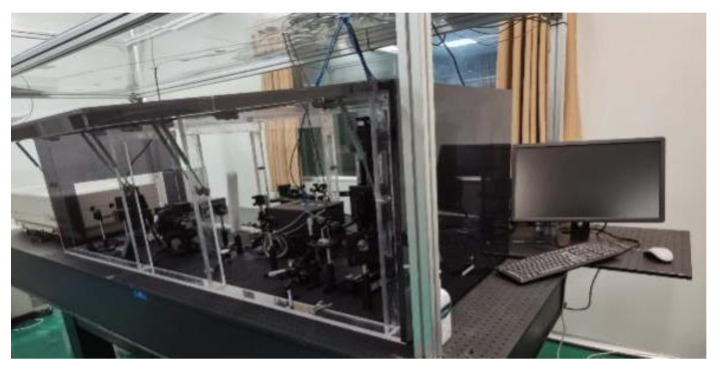
Damaged optics preparation platform.

**Figure 3:**
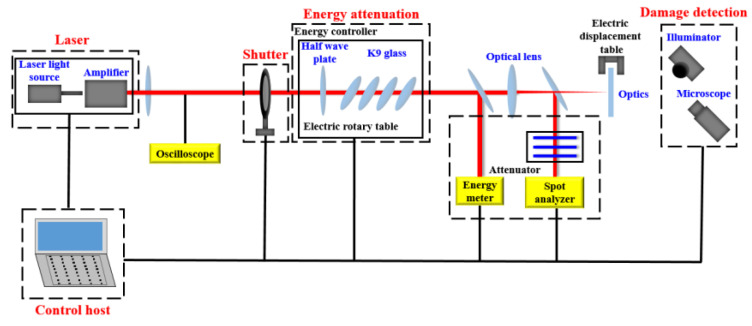
Laser damage test diagram.

In order to obtain fused silica samples with laser damage, the damage threshold test platform (Figure 2) was used to prepare the samples. The platform was built by our team, the light path and optics were maintained by Institute of Optoelectronic Technology, Harbin Institute of Technology twice a year. The test diagram is shown in Figure 3.

Combined with the service environment of fused silica optics in Shenguang device, we selected the laser with the same energy density and wavelength as that of the device to irradiate the fused silica optics many times to induce laser damage. During sample preparation, the laser wavelength was 355 nm, the pulse width FWHM (full width at half maximum) was 7 ns, the average output energy is 80 mJ, the spot area was about 1.5 mm^2^, and the energy density was about 5.4 J/cm^2^ (the energy density of Shenguang device was 5 J/cm^2^ during operation). The fused silica damage sample is obtained through laser scans performed three times.

### 2.2. Methods

#### 2.2.1. MRF Polishing Technique

**Figure 4:**
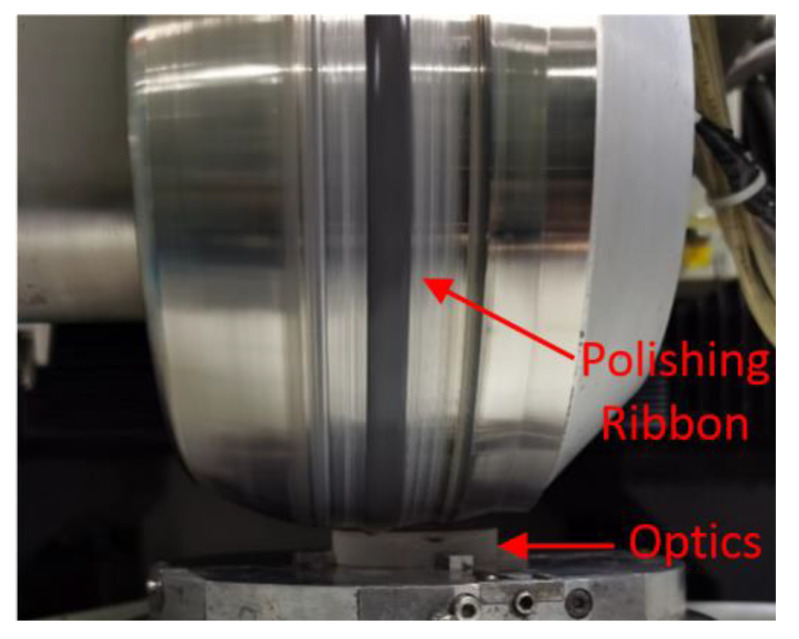
MRF polishing device.

In this work, the MRF technique was applied to repair small-scale laser damage on the surface of fused silica optics. The parameters in repair process are shown in Table 1, and the MRF device is shown in Figure 4.

#### 2.2.2. Laser Scattering Test

The surface defect laser scattering test was carried out on the laser scattering detection platform (ZC Co., Ltd., Shenzhen, China). The detection sensitivity was better than 0.5 μm, the temperature was 24 °C ± 2 °C and humidity was 40% ± 2%.

#### 2.2.3. Photothermal Absorption Test

The photothermal absorption was carried out on the photothermal absorption platform (ZC Co., Ltd.). The size of detection area was 5 mm × 5 mm, step length was 0.05 mm, pump power was 3.91 W, pulse repetition frequency (PRF) was 50 kHz, integration time was 300 ms, measurement mode was transmission, laser wavelength was 355 nm, sensitivity was better than 0.1 ppm.

## 3. Results

### 3.1. Damage Sample Preparation Result

**Figure 5:**
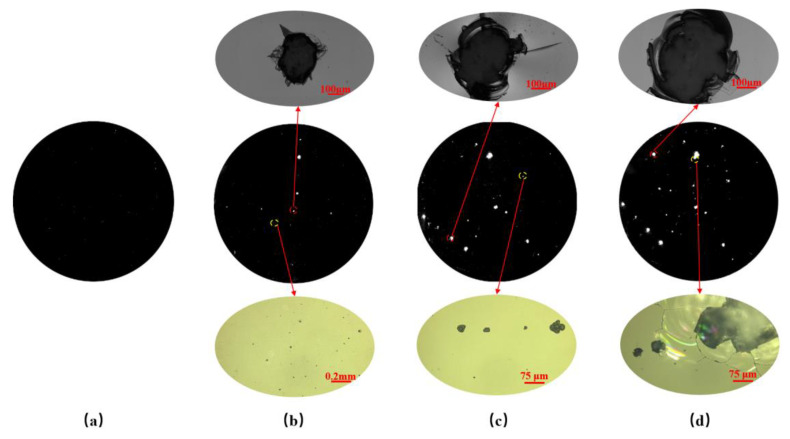
Laser scanning results: (**a**) Initial surface; (**b**) The result of first laser irradiation; (**c**) The result of second laser irradiation; (**d**) The result of third laser irradiation.

According to the parameters in Section 2.1 and the platform shown in Figure 2, the damage sample preparation was carried out. The surface of the optics was scanned by the laser three times. The laser scattering results are shown in Figure 5.

In Figure 4, it can be seen that a large number of laser-induced damage points with typical stress cracks and laser ablation occurred on the surface after laser irradiation three times. Along with the increase in laser irradiated times, not only did the number of damage points with different sizes change but so was the morphology of the laser damage. To accurately grasp the change in number, the defects of fused silica optics were counted and the results are shown in Table 2.

After laser irradiation, 602 laser damage points were generated on the surface, which was about three times the number of the defects of the initial surface. With the increase in laser irradiation times, the number of laser damage points increased gradually, which may indicate that the damage points generated by the previous laser irradiation will induce new ones. After counting the defects of defects on the irradiated surface, it can be found that the number of laser damage points (<50 μm) was 520, accounting for about 86% of the total number of laser damage points and for 77.2% of defects (<50 μm). According to the statistical results, the damage points were mainly small-scale (the diameter was less than 50 μm), so it was very important to remove the small-scale laser damage points during the whole repair process. If no special statement was made later, small-scale means the size <50 μm.

### 3.2. MRF Polishing Experiment of Damaged Optics

To preliminarily verify the effect of removing small-scale damage points, the MRF technique (technical parameters are shown in Table 1) was applied to polish the damaged samples prepared in Section 3.1, and the removal depth was set at 1.5 μm, 3 μm, 4.5 μm and 6 μm, respectively. A laser scattering defect detection platform was used to detect the surface before and after the MRF polishing process. The detection laser power was set to 800 mW.

**Figure 6:**
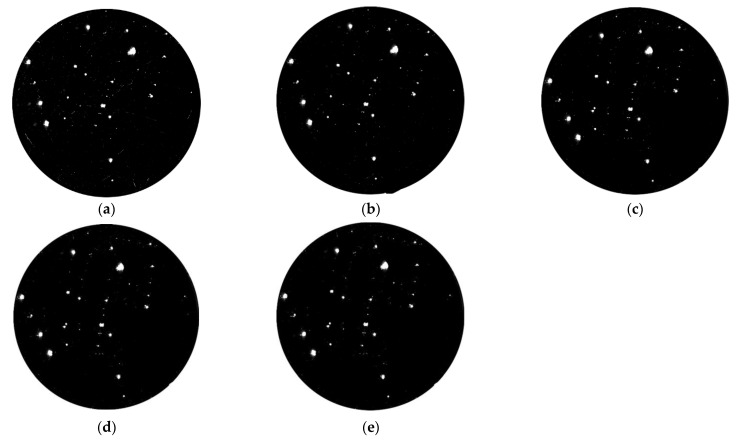
Laser scattering results after MRF process: (**a**) Material removal depth 0 μm; (**b**) Material removal depth 1.5 μm; (**c**) Material removal depth 3 μm; (**d**) Material removal depth 4.5 μm; (**e**) Material removal depth 6 μm.

In Figure 6, it can be found that the number of small-scale defects decreased significantly after the MRF polishing process, and the morphology of large-scale damage points changed slightly, while its number remained basically unchanged. According to the statistics on the number of small-scale defects (Table 3), the number of small-scale defects was reduced by 90% after removing materials for 4.5 μm. This removal depth could basically remove all small-scale laser damage points (number: 520). The defect suppression coefficient (90%) could be used as the target value for the MRF process, that is, when the number of defects is reduced by 90%, we believe that the small-size laser damage points generated during laser irradiation were completely removed. The above experiments also verified the effectiveness of the MRF technique in removing small-scale defects. It was worth noting that the number of small-size defects decreases rapidly at the initial stage of material removal, and was relatively slow at the later stage (the removal depth was 6 μm). We believe this relates to the morphology of some defects. Although the transverse size of these defects was small, they had a depth of several microns or even tens of microns, which limited the removal rate to a certain extent.

**Figure 7:**
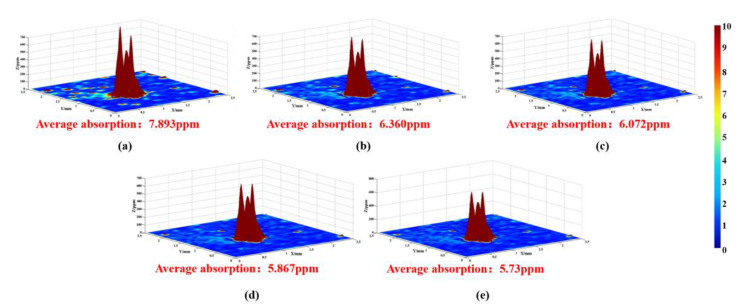
Photothermal absorption results after MRF process: (**a**) Material removal depth 0 μm; (**b**) Material removal depth 1.5 μm; (**c**) Material removal depth 3 μm; (**d**) Material removal depth 4.5 μm; (**e**) Material removal depth 6 μm.

Along with the laser scattering test, the photothermal absorption platform was also applied to detect the absorbed characteristics of optics in the MRF process, and the results are displayed in Figure 7. The testing parameters are listed in Section 2.2.3 and the test was in situ. The size of the test area was 2.5 mm × 2.5 mm.

In Figure 7, a lot of points with high absorption appeared in the testing area, which was linked to the existence of damage points. After removing materials for 1 μm, the absorption value decreased rapidly, from 7.893 ppm to 6.360 ppm, and some high-absorption points disappeared. With the increase in material removal, the absorption signal continued to decline, and the final signal came to 5.73 ppm. It should be noted that the signal decreased slowly during the MRF process, and a lot of high-absorption points were still maintained on the surface even with a material removal of 6 μm. In the early stage, the signal decreased rapidly, which was linked to the removal of small-scale defects. After removing materials for several microns, some defects with BD ratio (breadth depth ratio) still existed on the surface, which lead to a high-absorption signal.

### 3.3. Optimal Removal Depth Based on BP Neural Network

In the above section, it was verified that the MRF technique can effectively remove small-scale defects on the surface of damaged fused silica optics. In the removal process of laser damage at the present stage, machining and detection iteration were still applied in the repair process, which was time-consuming. For fused silica damaged optics, the number of small-scale damage points was generally different. If the MRF technique was used to remove materials of the same depth, two different results may appear: 1. Excessive material removal wasted processing time and reduced processing efficiency. 2. The amount of material removal was too small, and the small-size damage points were not removed sufficiently, resulting in invalid repair. Therefore, it was very important to accurately know the MRF polishing depth of each of the optics in the repair process.

In this section, the parameters of the MRF technique were optimized by BP neural network and the correlation between material removal depth and small-scale defects was established. Although the number of small-scale damage points of different optics was different, these damage points were generated under laser irradiation with the same wavelength, energy density and other parameters. Therefore, it could be considered that the characteristics of the initial damage points and the properties’ evolution in the MRF polishing process are basically consistent. The reliability of the material removal depth optimized by the BP neural network was high.

**Figure 8:**
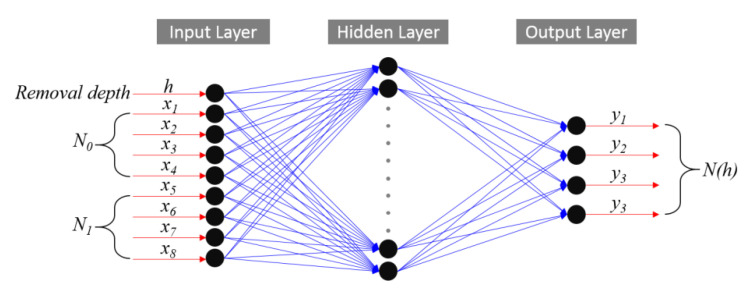
BP neural network structure diagram.

A BP neural network is a multilayer feed-forward network that optimizes the thresholds and weights of nodes through an error backpropagation algorithm. Generally, a BP neural network model included a three-layer network structure of one input unit, one or more hidden layers and one output unit. For general issues, it could be solved by a single hidden layer. Therefore, the model in this work mainly included an input layer, a hidden layer and an output layer [20,21]. As shown in Figure 8, assuming that the initial damage distribution of the damaged element was *N*_0_, and the damage distribution of material removal of 1 μm was *N*_1_, this model predicted the corresponding damage distribution *N_h_* of the through *N*_0_, *N*_1_ and removal depth *h*. The input unit included three parts *N*_0_, *N*_1_ and *h*, in which *N*_0_ was composed of *x*_1_ (the number of damage points (<50 μm)), *x*_2_ (the number of damage points (50 μm < × < 200 μm)), *x*_3_ (the number of damage points (200 μm < × < 400 μm)) and *x*_4_ (the number of damage points (>400 μm)), *N*_1_ was composed of *x*_5_ (the number of damage points (<50 μm)), *x*_6_ (the number of damage points (50 μm < × < 200 μm)), *x*_7_ (the number of damage points (200 μm < × < 400 μm)) and *x*_8_ (the number of damage points(>400 μm)), and the number of inputs was nine. The output of the model was *N_h_*, which consisted of *y*_1_ (the number of damage points (<50 μm)), *y*_2_ (the number of damage points (50 μm < × < 200 μm)), *y*_3_ (the number of damage points (200 μm < × < 400 μm)) and *y*_4_ (the number of damage points (>400 μm)), and the number of outputs was four. Therefore, this model could be expressed as

*N*_*h*_ (*y*_1_, *y*_2_, *y*_3_, *y*_4_) = *f* (*N*_0_ (*x*_1_, *x*_2_, *x*_3_, *x*_4_), *N*_1_ (*x*_5_, *x*_6_, *x*_7_, *x*_8_), *h*) (1)

For the neurons in the hidden layer, an empirical formula was used to make a preliminary judgment, and then the number was determined by comparing the error values. In this work, the number of input neurons was nine. According to the Kolmogorov theorem, the number of hidden neurons was about 19. In order to compare the training effect, the experiment under the number of hidden layer neurons 17 and 21 was also carried out at the same time.

**Figure 9:**
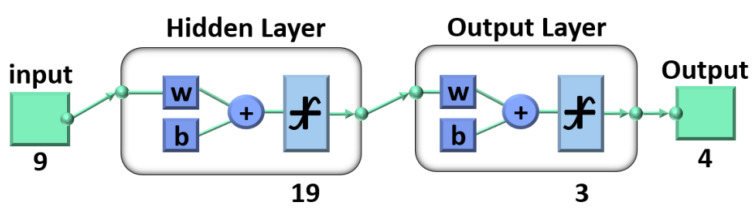
Design diagram of BP neural network model.

The training was carried out in the neural network toolbox of matlab 2019a, and the structural design is shown in Figure 9. The Tansig transfer function was selected for the hidden layer and the purelin transfer function was selected for the output layer.

In the early stage, 260 groups of damage points distribution data were prepared through the uniform removal experiment of damaged fused silica optics (number of elements: 10, processing times of a single sample: 10, single material removal amount: 1 μm), of which 190 groups were used for neural network training. The parameter settings for the training process were as follows: the number of display intervals was 100, the maximum number of cycles was 1000, the target learning error was 1, the learning rate was 0.002, and the number of hidden layer neurons was set to 17, 19 and 21, respectively. The convergence of model training error is shown in Table 4.

**Figure 10:**
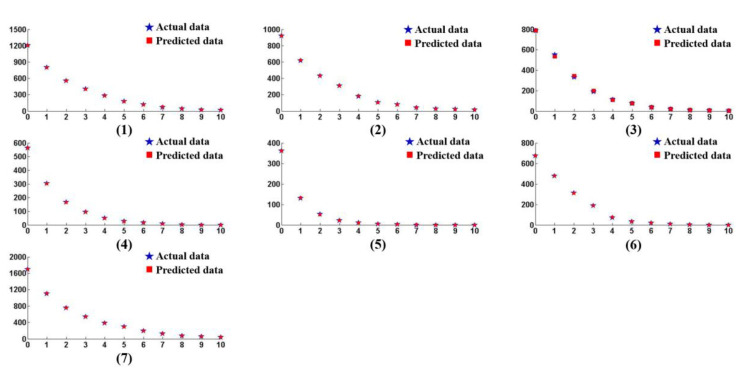
Predicted and actual number of damage points (<50 μm) under seven different sets of initial damage points.

According to Table 4, when the training steps reached 1000, the model errors of different numbers of neurons had basically converged to the target. By comparison, the error convergence was the best when the number of neurons was 19, therefore this value was applied in the hidden layer. Then, 70 groups of data that were not used for training were applied to the neural network model to obtain the distribution of small-scale defects with different removal depths. The predicted results and the actual test results are shown in Figure 10, and the relative error between the data is shown in Table 5.

**Figure 11:**
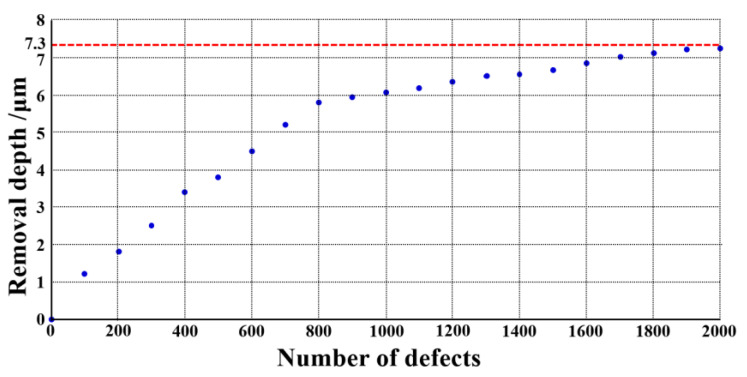
Predicted removal depth of different number of defects.

In Table 5, the relative errors between the predicted value and the actual value of the different experimental groups were not the same, and the errors also presented obvious differences within each group of data. From the results, it can be seen that the relative errors were basically limited to 5% when the removal depth was not enough to make the suppression coefficient reach 90%. In the early stage of the MRF process, the predicted value was equal to the real value because of the large number of normal small-scale defects, and the maximum of the errors was merely 5.12%. After the suppression coefficient exceeded 90%, the relative error gradually increased, even coming to 200%, which was linked to the defects with a larger BD ratio, as mentioned in Section 3.2. In this work, the target suppression coefficient was 90%, therefore the accuracy of the model was appropriate for this work and the prediction results could accurately guide the MRF polishing process. At the same time, according to the neural network model, we calculated the corresponding material removal depth when the inhibition rate of small-scale defects with different numbers reached 90%. The results are shown in Figure 11.

With the increasing number of small-scale defects, the corresponding removal depth also increased. When the number of defects was higher than 1800, the growth rate of the optimized removal depth gradually slowed down, and its value was basically maintained at about 7.3 μm. When the number of surface defects was small, the appropriate material removal depth could be selected according to Figure 7. When the number of surface defects was higher than 1600, the material removal depth could be set to 7.5 μm in order to remove all laser damage points stably.

### 3.4. Experimental Verification of Damage Repair

**Figure 12:**
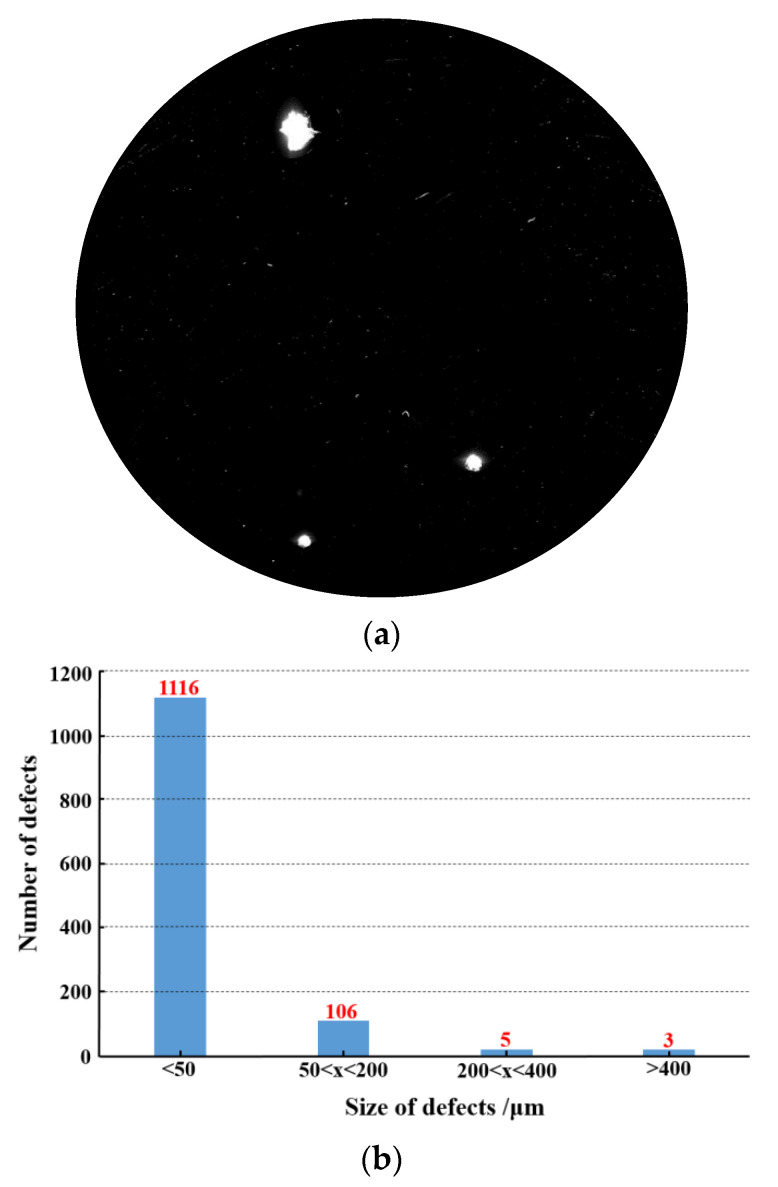
Results of surface defects before MRF polishing: (**a**) Laser scattering detection results; (**b**) The number of defects.

In order to examine the accuracy of the results in Section 3.3, a fused silica optic with a certain number of laser damage was polished by the MRF technique. Before processing, laser scattering detection was conducted on the surface to determine the number of surface defects. The results are shown in Figure 12.

**Figure 13:**
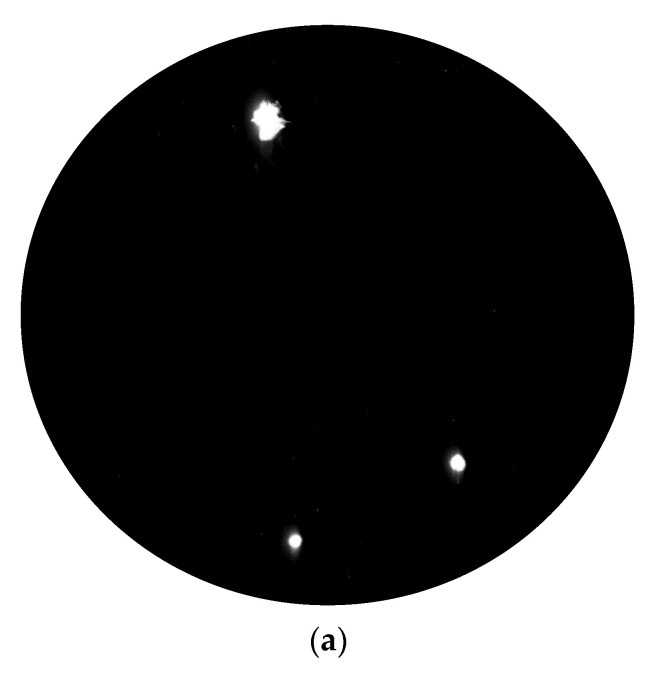

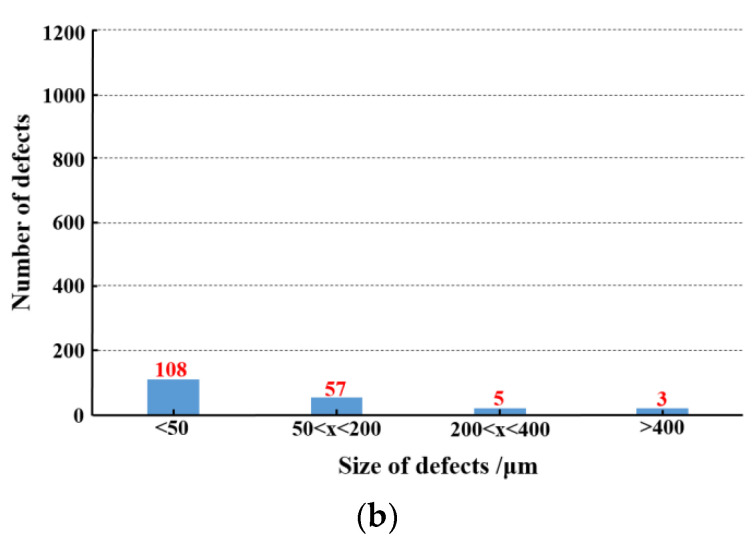
Results of surface defects after MRF polishing: (**a**) Laser scattering detection results; (**b**) The number of defects.

As shown in Figure 12, the number of small-scale defects of damaged fused silica optic was 1116. In combination with the results of Figure 11, the material removal depth of the MRF process was set to 6.2 μm, and other process parameters were the same as those in Table 1. The surface detection results after polishing are shown in Figure 13.

After the MRF polishing process, the number of surface defects greatly reduced, the number of small-scale defects (<50 μm) decreased from 1116 to 106, and the defect suppression coefficient was 90.32%, which reached the target as stated in Section 3.2. At the same time, the number of defects (50 μm < × < 200 μm) also decreased significantly, from 106 to 57. The number of defects with larger sizes had no obvious change before and after MRF polishing. It was believed that MRF polishing has poor inhibition ability for defects with larger scale (>200 μm), and a CO_2_ laser process could be used to passivate them later.

## 4. Discussion

In this work, a BP neural network combined with the MRF technique was used to suppress the defects within a certain scale on the surface of damaged fused silica optics and achieved remarkable results. However, there are still many phenomena to be discussed.

As shown in Section 3.1, the damage threshold test platform was used to prepare the damaged optics instead of using the optics banned from the Shenguang device. As for the real optics banned from the Shenguang device, their caliber is basically beyond 300 mm, and they have to be stored appropriately to avoid flowing to other institutions. Therefore, it is very difficult to obtain damaged optics from the device. The operating parameters of the Shenguang device can be obtained from relevant studies. Therefore, it is feasible to prepare the optics by ourselves. Except for the difference in the illuminance area of the laser, other operating parameters can be strictly controlled. The difference in laser will not strongly affect the damage types, the damaged optics prepared by the laser damage test platform can be applied in this work and the results still have a powerful reference value.

For the laser scattering device, which was developed by ZC Co., Ltd., the resolution and accuracy are high after being checked by the company. In Figure 5, Figure 6, Figure 12 and Figure 13, the damage points can be obviously found in the testing area, and the changes in the surface before and after the MRF process can also be easily obtained even though the size of the defects is very small (about a few microns). It should be noted that some testing results are in situ, which also verifies the accuracy. Based on the testing results and characteristics of the device, it can be proven that the device can be applied in this work and the testing results have strong credibility.

**Figure 14:**
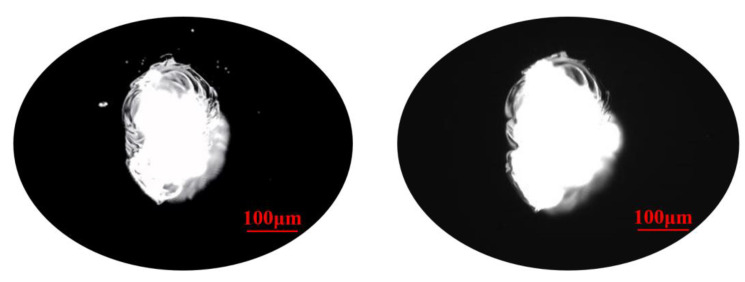
The morphology of large-scale defects before and after MRF process.

**Figure 15:**
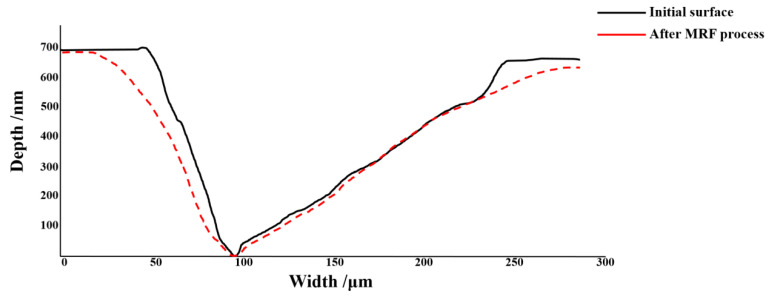
The section curve of large-scale defects before and after MRF process.

From Figure 5 and Table 3, it can be seen that the number of small-scale defects decreases rapidly, which indicates that small-scale defects are removed during the MRF process. As for large-scale defects, their number does not change obviously. In Figure 14, it could be seen that the small-scale defects around larger damage points were removed absolutely, and the large-scale damage point was mitigated. After the MRF process, the profile of the damage points became fuzzy, which was linked to the material removal, and the section curve (Figure 15) also verified the mitigation of the defects. In Figure 13, the width of damage points expanded slightly after the MRF process, from 250 μm to 280 μm, while the depth was basically maintained at about 700 nm. For large-scale damage points, the profile evolution represented a trend of mitigation, which was that the BD ratio increased. In the MRF process, the lateral dimension of large-scale expanded along with the material removal on the near-surface. However, the MR fluid piled up at the damage points, and nearly no material was removed inside the damage point, which caused the inapparent effect of mitigation.

As for the BP neural network model in this work, it plays an important role in the repair process of damaged fused silica, which can improve the processing efficiency. However, the profile characteristics of laser damage points were not taken into count, and the reasons were: 1. Different damage types. 2. The huge number of laser damage points. 3. Different damage profiles. 4. Some very special cases such as the defects with a high BD ratio. All these factors could not be input into the model totally, and that limits the integrity of the model. At the present stage, this model is appropriate for the repair process of small-scale damage points by the MRF technique. Along with the improvement of the methods of detection and characterization, more characteristics of the defects could be obtained, and the accuracy of the model will come to a new stage, even potentially universal to other repair techniques.

For the target of defect suppression, the standard value of 90% was selected in this work, which deviates from the ideal target (100%) of defect removal on the initial surface state. After the fabrication of optics, a certain number of small-size defects still remain on the surface. Dust and other pollutants may also be introduced in the process of loading and unloading. All the above factors result in a non-zero condition of defects on the initial surface, and the existence of defects will interfere with our judgment on the number of laser damage points. If the defects after laser irradiation are removed according to the 100% suppression coefficient, the material removal amount will increase greatly. Meanwhile, the inhibition of defects in the later stage of the MRF polishing process gradually decreases, which was linked to the conditions of defects and their distribution. Therefore, we believe that the selection of 100% inhibition efficiency is unreasonable in this work. Combined with the results in Table 2 and Table 3, it can be found that the number of defects after the MRF technique is lower than the number of defects in the initial state when the percentage of small-size defects on the surface of the element decreases by 90%. We believe that the damage points on the surface are completely removed at the same time, and the coefficient of 90% can be used as the target.

Secondly, due to a large number of small-scale defects, these cannot be effectively distinguished from the laser damage points. Therefore, it is impossible to accurately repair the laser damage. At present, the repair effect can only be judged by the change in the number of defects, which is another reason why the defect removal target is set to 90%. Finally, we also found that the inhibition effect of defects gradually decreased with the increase in removal depth, i.e., the number of defects decreased more slowly. Many damage points are still found on the surface under the ultra-high depth material removal for about 20 μm, which may mean that the defect suppression efficiency cannot reach 100%. In general, the setting of 90% defect suppression efficiency is appropriate in this work. For larger damage points (>50 μm), the changes in number in the MRF process were also displayed in this work, but they are not the goal of this paper. The suppression of this kind of defect will be described in the subsequent CO_2_ laser repair work. The evaluation objective of the repair effect is not based on the suppression coefficient any longer, but more on the optical properties of the repair points.

## 5. Conclusions

As an optical material with excellent properties, fused silica is widely used in the manufacturing process of optics applied in various optical systems. Aimed at the damaged optics in high-power laser systems, the MRF suppression method for small-scale damage points was studied in this work. Through a BP neural network, the optimal removal depth corresponding to different initial damage states was optimized. Based on a BP neural network model, the MRF polishing experiment was carried out, and the advantages of the MRF technique in the removal of small-size damage points were verified. The distribution and the number of small-scale damage points after processing basically met the requirements of re-shelving. The method proposed in this work cannot only shorten the processing time and improve the processing efficiency but also avoid the waste of fused silica material, which has a certain application value and economic value. The relevant theoretical models and processing methods can provide guidance for the repair of fused silica-damaged optics.

## Figures and Tables

**Table 1:** Parameters of MRF repair technique.

Parameters	Value
Polishing wheel speed	280 r/min
Flow rate	120 L/min
Electricity	8 A
Pressed depth	0.25 mm
Abrasive type	CeO_2_

**Table 2:** Statistics of defects with different scales.

Irradiation Times	Defects (<50 μm) Number	Defects (50 μm < × < 200 μm) Number	Defects (200 μm < × < 400 μm) Number	Defects (>400 μm) Number	Damage Growth Number
0 (initial)	153	47	2	0	0
1	255	55	8	6	122
2	404	75	22	12	189
3	673	86	21	24	291

**Table 3:** Defects number with different removal depths.

	Initial Surface	Removal Depth 1.5 μm	Removal Depth 3 μm	Removal Depth 4.5 μm	Removal Depth 6 μm
Defects number	673	307	180	105	78

**Table 4:** Error convergence of different neurons in hidden layers.

Number of Neurons: 17		Number of Neurons: 19		Number of Neurons: 21	
Steps	Error	Steps	Error	Steps	Error
200	0.04756	200	0.03854	200	0.04325
400	0.01453	400	0.00959	400	0.01103
800	0.00456	800	0.00215	800	0.00359
1000	0.000973	1000	0.000431	1000	0.000758

**Table 5:** The relative error between predicted results and the actual test results.

Removal Depth/μm	Errors of Result (1)	Errors of Result (2)	Errors of Result (3)	Errors of Result (4)	Errors of Result (5)	Errors of Result (6)	Errors of Result (7)
1	0.748%	0.97%	3.26%	0.98%	1.53%	0.42%	0.18%
2	0.358%	0.46%	3.02%	0.6%	4%	0.32%	0.26%
3	1.47%	0.64%	3.15%	1.05%	0% (*)	0%	0.186%
4	0.714%	1.11%	3.57%	2% (*)	10%	1.39%	0.261%
5	0.568%	1.92%	2.63%	3.84%	25%	3.03% (*)	0.34%
6	1.69% (*)	5.12% (*)	0% (*)	5.8%	50%	11.1%	1.05%
7	2.816%	4.76%	5.26	12.5%	100%	12.5%	0.819% (*)
8	4.76%	11.1%	11.1%	0%	0%	0%	1.38%
9	0%	18.1%	50%	0%	0%	0%	1.87%
10	5.88%	12.5%	200%	0%	0%	0%	7.14%

Explanation: The symbol of “(*)” represent the removal depth when the suppression coefficient of defects came to 90%.
